# Research of Herb-Partitioned Moxibustion for Primary Dysmenorrhea Patients Based on the LC-MS Metabonomics

**DOI:** 10.1155/2015/621490

**Published:** 2015-07-01

**Authors:** Yu-xia Ma, Xing-yue Yang, Gang Guo, Dong-qing Du, Yan-pu Yu, Shu-zhong Gao

**Affiliations:** ^1^Shandong University of Traditional Chinese Medicine, Shandong 250355, China; ^2^Qilu Hospital of Shandong University, Shandong 250012, China; ^3^Jinan Hospital of Traditional Chinese Medicine, Shandong 250012, China

## Abstract

*Objective*. To explore the efficacy and mechanism of primary dysmenorrhea patients were treated with herb-partitioned moxibustion through metabonomics. *Methods*. 20 patients with primary dysmenorrhea were randomized into two groups, separately treated with herb-partitioned moxibustion at CV8 (*shenque*) and acupuncture at SP6 (*sanyinjiao*). After three menstrual cycles' treatment, the intensity of menstrual pain using VAS and the changes of metabolites of plasma using LC-MS were observed. *Results*. The VAS of two groups decreased with different descending range. Herb-partitioned moxibustion upregulated 20*α*-dihydroprogesterone, pregnenolone, prostaglandin E_2_ and *γ*-aminobutyric acid and downregulated the content of estrone and prostaglandin H_2_, while acupuncture upregulated pregnenolone and 20*α*-dihydroprogesterone and downregulated 2-methoxyestradiol-3-methylether, 15-hydroxyeicosatrienoic acid and 6-keto-prostaglandin. *Discussion*. It was effective in relieving the abdominal pain by these two therapies. Herb-partitioned moxibustion is superior to acupuncture for primary dysmenorrhea, which could be related to regulating the endocrine hormone.

## 1. Introduction

Primary dysmenorrhea (PD) is the painful menstrual cramps of uterine in woman during menstruation without detectable organic disease [1]. Most of the PD patients develop the typical symptoms such as crampy pelvic pain with pain radiating to the lower back or anterior thigh, nausea, vomiting, diarrhea, headache, fatigue, nervousness, and dizziness before or at the onset of menses and lasting one to three days [2]. It is the most common gynecologic complaint especially among adolescent women with estimates ranging from 40 to 50 percent [3], which has disturbed their routine activities and may lead to infertility in severe cases [4, 5]. The main treatment of modern medicine is oral spasmolysis analgesic, acyeterion, and calcium ion antagonist. Nowadays, nonsteroid anti-inflammatory drug (NSAIDs), like Ibuprofen, indomethacin, and chlorine acid, is the first-line drugs for the treatment of primary dysmenorrhea [6], which is to reduce the accumulation of prostaglandin and then alleviate spasmodic contraction of the uterus caused by prostaglandins, by inhibiting the COX-2 enzymes activity [7]. But at the same time COX-1 would also be inhibited and then causes adverse reactions such as nausea, diarrhea, and headache [8]. Recent studies have shown that cardiovascular and cerebrovascular diseases would also be induced through chronic use of these drugs, such as unstable angina, acute myocardial infarction, and venous thromboembolism [9].

Therefore, it is necessary to seek a new effective therapy to treat PD. Traditional Chinese medicine (TCM), such as acupuncture [10] and herbal medicine [11], has been evaluated through a number clinical trials, suggesting preliminary evidence of efficacy for PD. Herb-partitioned moxibustion (HPM) is a characteristic external therapy in TCM, which is widely used to treat diseases in China. The preliminary work of our research team has proved that HPM can immediately relieve menstrual pain [12]. But its mechanism is still unclear. Therefore, it needs further study.

Metabonomics indicates the overall physiological status by analyzing the dynamic changes of low molecular weight metabolites [13]. The pathogenesis of diseases and the action mechanisms of therapy would be elucidated by identifying the biomarkers and analyzing the metabolic pathway. Recently, metabonomics has attracted an interest for biomarker discovery and for assessing holistic therapeutic effects of many TCMs [14, 15]. Liquid chromatography-mass spectrometry (LC-MS) has demonstrated being the frequently used analytical technologies in metabonomics-based studies [16, 17], allowing expanding the number of metabolites that can be comprehensively covered in experiments.

In this study, our aims are (1) to verify and to compare the clinical efficacy of HPM and acupuncture for PD patients; (2) to preliminarily elucidate the possible mechanisms of HPM for PD by using a metabonomics analysis.

## 2. Methods

### 2.1. Patient Selection

Patients were recruited from the outpatients of Affiliated Hospital of Shandong University of Traditional Chinese Medicine between January 2011 and December 2012. The study protocol was approved by the Ethics Committee of Shandong University of Traditional Chinese Medicine Affiliated Hospital in 2010 (Registration number 20100137). All the volunteers provided informed consent.

Eligible patients met the following inclusion criteria: (1) the diagnostic criteria of primary dysmenorrhea in the Primary Dysmenorrhea Consensus Guideline [18]; (2) age from 16 to 35 years without history of delivery; (3) being with normal menstrual cycle (28 ± 7 days); (4) VAS (visual analogue scale) scoring more than 40 mm; (5) no oral administration of any analgesic and other hormones such as oral contraceptive pill or acceptance of other therapies on menstrual cycle before the trial and during the experimental period. The following main criteria for exclusion were applied: women with secondary dysmenorrhea caused by endometriosis, uterine myoma, endometrial polyps, pelvic inflammatory disease, and other gynecological problems; pregnant and lactating women; women with other serious illnesses and unsuited for acupuncture.

### 2.2. Randomization and Blinding

**Figure 1:**
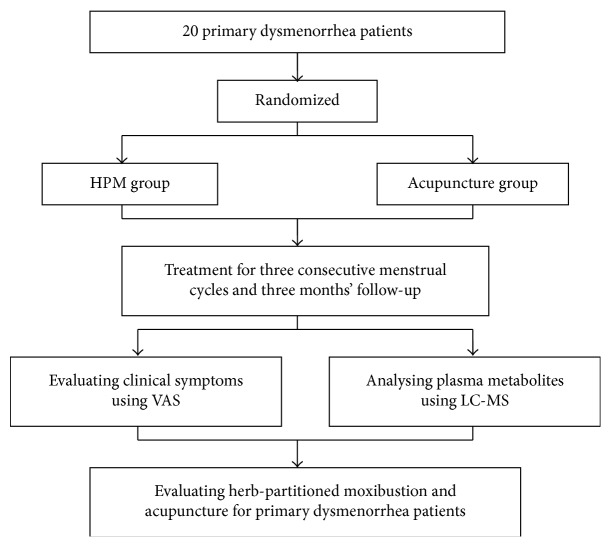
Flow chart of the study.

Randomization was performed by an independent statistician through generating allocation numbers based on a random number creation system. 20 eligible patients were randomly assigned into two groups (HPM group and acupuncture group), each group with 10 patients, respectively (Figure 1). Double-blind trial could not be performed in this study. However, data collectors and data statisticians were all blinded to treatment assignments.

### 2.3. Interventions

The points used in this study were located according to Chinese National Criteria for Points Location (GB12346-90). Acupuncture interventions were performed by the same acupuncturist who had the Chinese medicine practitioner license from the Ministry of Health of the People's Republic of China throughout the entire study. Sterile disposable acupuncture needles (*Huatuo* 0.30 × 40 mm, made in* Suzhou* Medical Instruments Factory) were used. The medicinal herbs used were purchased from* Jianlian* Medicine Company (*Jinan*, China) and were authenticated by professor of pharmacognosy. The herbs mainly included* wuzhuyu, bai shao, ruxiang, moyao, yanhusuo, bing pian*, and* wulingzhi*, which were smashed by ultrafine grinder into pulverata.

#### 2.3.1. HPM Group

**Figure 2:**
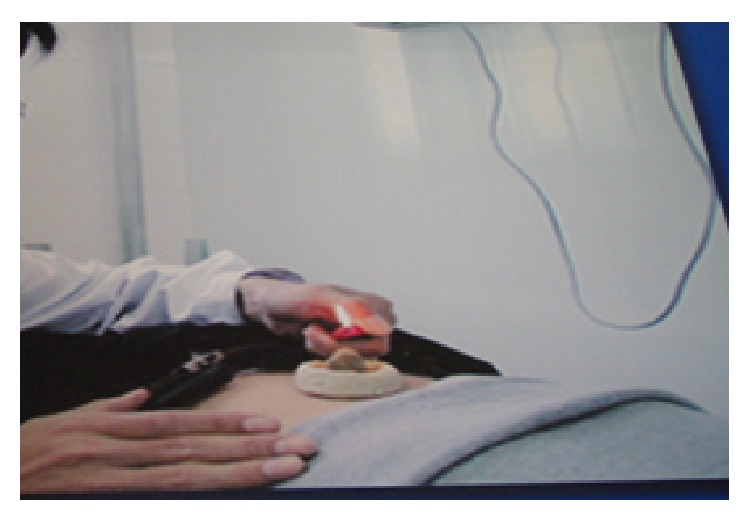
HPM for PD patients in clinic. A bowl made by dough with a hole (diameter 2 cm, depth 2 cm) in the middle was placed on CV8. Medicamental pulverata (about 8–10 g) was filled in the hole. Then a burning moxa cone (diameter 2 cm, height 2 cm) was put on the medicamental pulverata and changed till it burned out. Ten moxa cones were used, about 2 hours, during each treatment time.

The herbs were mixed in proportions and were shattered into medicamental pulverata by pulverizer. Participants were asked to lay on their back, and then the CV8 (*shenque*, in the middle of navel) and its surrounding skin were disinfected using 75% alcohol. A bowl made by dough with a hole (diameter 2 cm, depth 2 cm) in the middle was placed on CV8. Medicamental pulverata (about 8–10 g) was filled in the hole. Then a burning moxa cone (diameter 2 cm, height 2 cm) was put on the medicamental pulverata and changed till it burned out (Figure 2). Ten moxa cones were used, about 2 hours, during each treatment time. At the end of the treatment, the medicamental pulverata was sealed with adhesive tape and washed 24 hours later.

#### 2.3.2. Acupuncture Group

The participants were punctured perpendicularly at bilateral SP6 (*sanyinjiao*, 3* cun* above the medial malleolus, at the posterior border of the medial aspect of the tibia) to a depth of 2.5–4.0 cm in supine position. Manipulating needling, lifting-thrusting and twirling for one minute, were used to induce* deqi*. And then the needles were retained for 15 minutes.

The performer started the intervention 7 days before menses once a day by acupuncture and once every three days by moxibustion until menstruating. Participants received the treatment for three consecutive menstrual cycles and three months' follow-up. After the course, we analyzed the outcomes. During the treatment period, the participants could take aspirin effervescent tablets orally distributed by performers when they could not endure menstrual pain (VAS ≥ 80), which would be recorded by the participants and dosage by the performers.

### 2.4. Outcome Measures

#### 2.4.1. The Primary Outcome Measures

The VAS was used to measure the abdominal pain of participants from baseline to follow-up recorded by another performer.

#### 2.4.2. The Secondary Outcome Measures

We utilized LC-MS to analyse plasma metabolites. 20 participants waited for one menstrual cycle without treatment before cycle 1. Blood specimens were collected in the first or second day during the menstruation of the waiting month and the first month after treatment course. The blood specimen was put into anticoagulant tube for centrifugation for 5 min with 5000 r·min^−1^ and then the plasma was kept at −28°C in reserve. The plasma specimen would be taken out before the testing and room temperature melting and then was processed with centrifugation for 5 minutes with 10800 rpm after being precipitated by acetonitrile first. Take the supernatant liquid for testing. LC-MS analysis was performed on Agilent 1290 Infinity LC system coupled to Agilent 6530 Accurate-Mass Quadrupole Time-of-Flight (Q-TOF) mass spectrometer (Agilent, USA). Chromatographic separations were performed on an ACQUITY UPLCTM BEH C18 column (2.7 *μ*m, 3.0∗50 mm), flow rate: 0.4 mL/min, temperature in the injection chamber: 4°C, column temperature: 35°C, and sample size: 5 *μ*L. An electrospray ionization source (ESI) interface was used and was set in both positive and negative modes so as to monitor as many ions as possible. The following parameters of MS were employed: electrospray ion source, mass scan range: 50–1100 Da, capillary voltage: 3500 V, quadrupole rod temperature: 100°C, fragmentor: 175 V, nebulizer pressure: 35 psig, gas temperature: 350°C, and gas flow: 10 L/min.

### 2.5. Statistical Analysis

The raw LC-MS data from metabolic profiling were pretreated following the procedure described. We analyzed the data using Masshunter quantitative data analysis software to extract molecular peak feature of data after removing noise, in addition to the internal standard, peak, and peak alignment, normalized, and then using Simca-P (version 11.5) software to proceed principal component analysis (PCA) and partial least square-discriminant analysis (PLS-DA), and applying VIP (very important in the projection) data to find out the characteristic metabolites. The related metabolic pathways information was from the HMDB and KEGG software. We used SPSS (version 17.0) to analyse the data; measurement data was presented by mean and standard error (SE), using *t*-test. *P* values reported in this paper are two-sided and *P* values of <0.05 were considered statistically significant.

## 3. Results

### 3.1. Menstrual Abdominal Pain with VAS Scores

**Figure 3:**
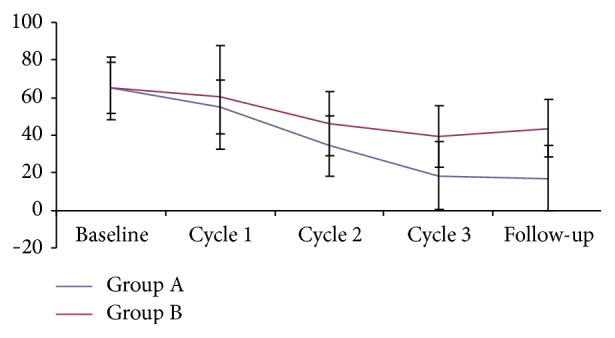
The VAS scores of each cycle.

From Figure 3, we could see that the VAS value of both two groups reduced gradually, and there was statistical significance compared to cycle 2, cycle 3, and follow-up with the baseline (*P* < 0.01). The comparison of VAS score between the two groups showed that there was statistical significance at cycle 3 and follow-up (*P* < 0.01). No difference was identified between Group A and Group B at the baseline, cycle 1, and cycle 2 (*P* > 0.05).

### 3.2. Analysing the Potential Biomarkers

**Figure 4:**
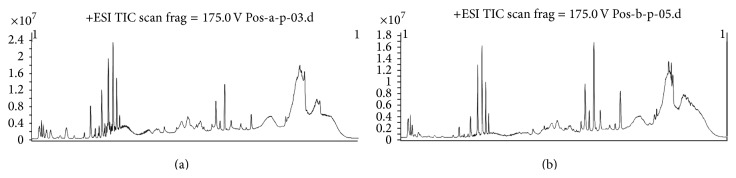
The TIA of two groups after treatment in ESI^+^ mode: (a) HPM group; (b) acupuncture group. Group HPM and group acupuncture had obvious differences.

**Figure 5:**
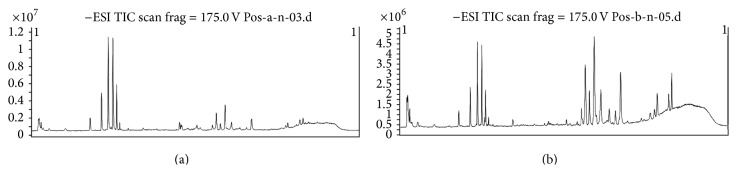
The TIA of two groups after treatment in ESI^−^ mode: (a) HPM group; (b) acupuncture group. Group HPM and group acupuncture had obvious differences.

**Figure 6:**
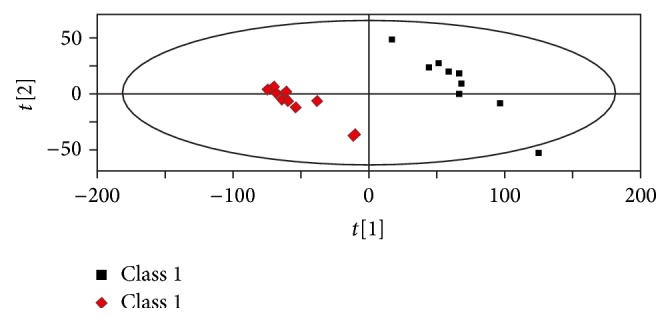
The PLS-DA plot from group HPM before and after treatment. Class 1: before treatment; class 2: after treatment.

**Figure 7:**
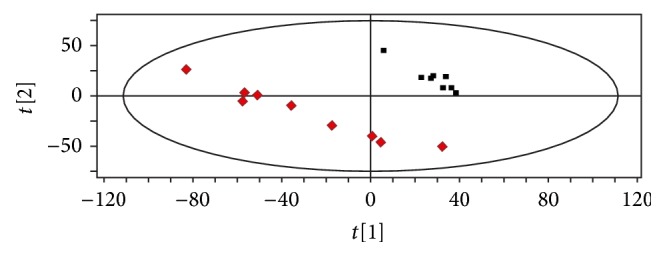
The PLS-DA plot from group acupuncture before and after treatment. Class 1: before treatment; class 2: after treatment.

The metabolomics figures of two groups were shown from Figures 4–7. Figures 4 and 5 show typical LC-MS total ion current (TIC) chromatograms of a plasma sample in positive ionization mode (Figure 4) and negative ionization mode (Figure 5). Compared to Figures 4(a) and 4(b), Figures 5(a) and 5(b), the TIC of group HPM and group acupuncture had obvious differences. In the PLS-DA plots (Figures 6 and 7), these two groups could also be well distinguished. Therefore, we deduced that the metabolite changes after treatment were different.

**Figure 8:**
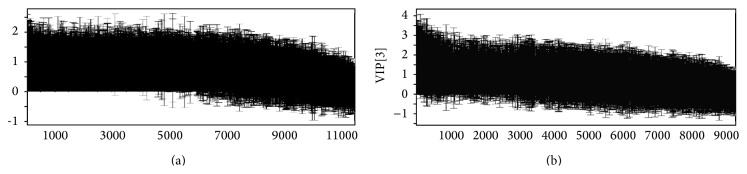
The VIP scores of PLS of two groups: (a) HPM group; (b) acupuncture group. The variation trend of metabolite, reducing from left to right (*P* < 0.05).

Figure 8 expressed the variation trend of metabolite, reducing from left to right. With VIP >1 as a standard (where a VIP value of >1 is regarded as significant), we found 6950 kinds of ion information of group HPM; 7 metabolites were considered as the potential biomarkers. In group acupuncture, we found 4460 kinds of ion information, and 5 metabolites were considered as the potential biomarkers. It was shown in Table 1. In this study, the HPM upregulated the 20*α*-dihydroprogesterone, pregnenolone, prostaglandin E_2_, and gamma-aminobutyric acid and downregulated estrone, 16-oxoestrone, and prostaglandin H_2_. Acupuncture upregulated pregnenolone, and 20*α*-dihydroprogesterone and downregulated 2-methoxyestradiol-3-methylether, 15-hydroxyeicosatrienoic acid, and 6-keto-prostaglandin. However, the remaining biomarkers (data not shown) were unidentifiable due to insufficient intensity for MS experiments or the restrictions of current metabolite databases.

## 4. Discussion

In the study, these results suggested that HPM could improve the menstrual pain of PD. LC-MS was used to investigate the plasma metabolic profile associated with PD. Seven potential biomarkers (20*α*-dihydroprogesterone, pregnenolone, prostaglandin E_2_, estrone, 16-oxoestrone, and prostaglandin H_2_) connected with PG, estrogen, and progestin have been found. The cramps of uterine in PD are thought to be caused by excessive production of prostaglandins (PG) and leukotrienes, which mediate hyperalgesia and cause vasoconstriction, ischemia, and myometrial contraction [19]. Moreover, the excess of PG would lead to cardiovascular and gastrointestinal symptoms [20]. It is indicated that the abnormal level of estrogen/progestin and immunologic function would lead to an excess of PG [21, 22]. Further investigating the intervening mechanisms of HPM for PD, we found that most of the potential biomarkers involved in metabolic processes were related to steroid hormone biosynthesis. In other words, HPM can regulate the internal secretion of PD patients, which is mostly compatible with the results of our previous study [12]. In previous study, we have found that patients with HPM showed significant differences after treatment in the level of estradiol, progesterone, and prostaglandin using chemoimmunology therapy. In addition, another potential biomarker, gamma-aminobutyric acid, takes part in analgesia in central nervous system that can increase the pain threshold. Therefore, gamma-aminobutyric acid increasing could alleviate pain. Furthermore, HPM can regulate arachidonic acid metabolism, producing PG and leukotrienes through the function of cyclooxygenase and lipoxygenase.

According to statistics, SP6 (*sanyinjiao*) is the most common selected acupoint in PD trials [23]. Therefore, we choose acupuncture at SP6 as the control group to demonstrate the clinical efficacy of HPM. From Figure 3, HPM has better curative effect in reducing the menstrual pain of PD patients, with stable efficacy in follow-up of three months. Meanwhile, the metabolite profiles of SP6 group showed 5 potential biomarkers (2-methoxyestradiol-3-methylether, 20*α*-dihydroprogesterone, pregnenolone, 15-hydroxyeicosatrienoic acid, and 6-keto-prostaglandin), three of which are the same as HPM group. One of the other two potential biomarkers, 2-methoxyestradiol-3-methylether, is also related with steroid hormone biosynthesis. And 15-hydroxyeicosatrienoic acid revealed metabolic pathway (arachidonic acid metabolism) is mentioned above in group HPM. Therefore, the regulating internal secretion might also be involved in the intervening mechanism of acupuncture at SP6 for PD.

Compared with HPM and acupuncture at SP6 for PD, the VAS score shows that both two therapies can relieve menstrual pain, but the former one has better clinical efficiency. Using LC-MS, we find the two therapies express the changes mainly of endocrine hormone to regulate the internal secretion. But HPM has wider regulation than acupuncture comparing the metabolites. The group of HPM is better than the group of acupuncture at SP6 in regulating the estrone and prostaglandin level. Through our previous work, CV8 (*shenque*) is associated with five* zang* and six* fu* according to TCM theory. It is regarded as congenital foundation and the pivot of* yin-yang* balance. It is also close to pelvic cavity in anatomical position. Furthermore, it is easy for the effective constituent of herbs penetrating into body due to the thin skin around it. The main efficacy of the herbs used in our study is warming and activating meridian, promoting* qi* circulation to relieve pain. The heat stimulation of moxibustion could accelerate the penetration of the herbs into human body. Therefore, HPM, combining the heat stimulation, herbal stimulation, and acupoint stimulation together, is suitable for gynecological diseases especially functional diseases such as PD.

However, whether HPM is superior to acupuncture or not is not concluded in this study because of a small sample experiment. It merits further study. Besides, on account of the metabonomics method, the metabolism of the whole body cannot be reflected fully. The further study on the mechanisms of the changes of metabolite of PD patients should expand the sample size and then, combined with molecular biology, imageology and other research methods to explore the mechanism of acupuncture and moxibustion for PD.

## 5. Conclusion

The present study plays an important role in providing an effective alternative for the treatment of PD and further investigating the mechanisms of HPM for PD. HPM can relieve menstrual pain of PD patients possibly through regulating internal secretion. On account of the present limitation, further study is being carried out in our laboratory.

## Figures and Tables

**Table 1:** The main probable materials of two groups.

Group	ESI	Monoisotopic molecular weight	Formula	Metabolites	Related pathway	VIP	Changed fold^a^
A	+	270.1620	C_18_H_22_O_2_	Estrone	Steroid hormone biosynthesis	1.56605	↓
A	+	316.2402	C_21_H_32_O_2_	20*α*-Dihydroprogesterone	Steroid hormone biosynthesis	1.30279	↑
A	+	316.2402	C_21_H_32_O_2_	Pregnenolone	Steroid hormone biosynthesis	1.30279	↑
A	+	352.2250	C_20_H_32_O_5_	Prostaglandin E_2_	Steroid hormone biosynthesis	1.47588	↑
A	+	352.2250	C_20_H_32_O_5_	Prostaglandin H2	Arachidonic acid metabolism	1.47588	↓
A	+	103.0633	C_4_H_9_NO_2_	Gamma-Aminobutyric acid	GABAergic synapse	1.3201	↑
A	−	284.1412	C_18_H_20_O_3_	16-Oxoestrone	Steroid hormone biosynthesis	1.10038	↓
B	+	316.2038	C_20_H_28_O_3_	2-Methoxyestradiol-3-methylether	Steroid hormone biosynthesis	1.59796	↓
B	+	316.2402	C_21_H_32_O_2_	20*α*-Dihydroprogesterone	Steroid hormone biosynthesis	1.12801	↑
B	+	316.2402	C_21_H_32_O_2_	Pregnenolone	Steroid hormone biosynthesis	1.12801	↑
B	+	322.25	C_20_H_34_O_3_	15-Hydroxyeicosatrienoic acid	Arachidonic acid metabolism	1.47681	↓
B	−	370.2356	C_20_H_34_O_6_	6-Keto-prostaglandin	Arachidonic acid metabolism	2.46181	↓

^a^compared to the content before treatment. Arrow (↑) indicated relative increasing in signal. Arrow (↓) indicated relative decreasing in signal. Differences with *P* < 0.05 were considered significant.
